# Effects of compression running pants and treadmill running stages on knee proprioception and fatigue-related physiological responses in half-marathon runners

**DOI:** 10.3389/fphys.2022.1035424

**Published:** 2022-12-05

**Authors:** Lin Chang, Silin Fu, Jianghua Li, Sam Wu, Roger Adams, Jia Han, Chunying Han

**Affiliations:** ^1^ School of Exercise and Health, Shanghai University of Sport, Shanghai, China; ^2^ College of Physical Education and Sports, Beijing Normal University, Beijing, China; ^3^ Department of Physical Education, University of International Business and Economics, Beijing, China; ^4^ Department of Health and Medical Sciences, Swinburne University of Technology, Melbourne, VIC, Australia; ^5^ Research Institute for Sport and Exercise, University of Canberra, Canberra, VIC, Australia; ^6^ College of Rehabilitation Sciences, Shanghai University of Medicine and Health Sciences, Shanghai, China; ^7^ School of Arts, Shanghai University of Sport, Shanghai, China

**Keywords:** half-marathon, compression running pants, knee proprioception, physiological, treadmill running

## Abstract

**Background:** Knee injury is common in half-marathon runners, however, the effect of compression running pants on fatigue and knee proprioception remains unclear.

**Objectives:** The study aims to investigate whether wearing compression running pants (CRP) and treadmill running stages affect knee proprioception and fatigue-related physiological responses during half-marathon running.

**Methods:** Eighteen half-marathon runners completed two self-paced 21 km treadmill running trials, once wearing CRP and once wearing loose running shorts (LRS). For each 21 km run, RPE, heart rate, blood lactic acid, and knee flexion proprioception were assessed before starting, and after each 7 km stage.

**Results:** Data analysis revealed no difference between CRP and LRS conditions in heart rate, RPE, or blood lactic acid. Repeated measures ANOVA showed a significant garment condition main effect whereby wearing CRP was associated with higher knee proprioceptive acuity (*p* = 0.006). Polynomial trend analysis showed a significant linear downwards trend in proprioceptive acuity across the four measurement occasions (*p* = 0.048). Stage analysis showed that wearing CRP was associated with better knee proprioception at running distances of 14 km (*p* = 0.007, 95%CI = -0.054, -0.010) and 21 km (*p* = 0.016, 95%CI = -0.051, -0.006).

**Conclusion:** Compression running pants provide an overall positive effect on knee proprioception, particularly after 14 km and 21km, which may reduce the probability of knee injury. CRP had no significant effect on physiological measures in half-marathon running.

## Introduction

In recent years, the number of marathon events conducted around the world has increased such that the half marathon has become the sports event with the largest number of runners in the world (Knechtle et al., 2016). However, most runners lack knowledge and guidance regarding the prevention of injury, and there has been an associated increase in injuries associated with the event (Kakouris et al., 2021). The highest percentage of injuries occur in runners’ lower limbs, especially in the ankle and knee joints (Van Gent et al., 2007). The main types of injury were Achilles tendinopathy, patellar pain syndrome, iliotibial band syndrome, medial tibial stress syndrome and plantar fasciitis (Kakouris et al., 2021). One recent study found the injury rate at the knee joint in half-marathon runners to be 35% (Mohseni et al., 2021). Such a high injury rate imposes a severe psychological and financial burden on runners and has a detrimental socioeconomic impact (Hespanhol Junior et al., 2016). Therefore, it is essential to explore measures that could reduce knee injuries during half marathon running.

Increased lower limb injury risk in runners has been argued to be related to having lower levels of proprioception (Han et al., 2015). Proprioception is usually described as a sense of body position and movement (Gandevia et al., 1992), generated from proprioceptive mechanoreceptors located in the skin, muscles, tendons, and joint capsule as well as in the ligaments (Miura et al., 2004). Joint and tendon mechanoreceptors are complementary in proprioceptive acuity and their activation depends on knee stimulation (Lephart et al., 1998). In addition, because proprioceptive information from the skin and muscles is imperative for proprioception, enhancing tactile input may facilitate better neuromuscular control of the knee (McCloskey and Gandevia, 1978; Edin, 2001). Therefore, finding a way to stimulate proprioceptive input from the soft tissue around the knee may potentially increase proprioception and thus reduce knee injury rates.

In recent years, compression garments (CG) have grown in popularity among athletes (Broatch et al., 2020). Studies have shown that CG can improve sports performance, accelerate recovery from fatigue (Goh et al., 2011), and prevent injuries (Van Tiggelen et al., 2008; Fu et al., 2013). These effects may be because wearing compression garments might promote stable muscle alignment (Davies et al., 2009), enhance cutaneous sensory input (Herrington et al., 2005; Van Tiggelen et al., 2008), encourage the recruitment of nerve fibers in the muscles (Miyamoto et al., 2011), and reduce the spread of potentially harmful vibration damage in the muscles (Macefield, 2005; Jahjah et al., 2018).

In terms of their effects on proprioceptive performance in lower limb joints, Cameron et al. (2008) found that wearing compression shorts could improve hip extension proprioception in football players who performed poorly when not wearing them. In addition, a recent study found that wearing graduated compression socks could maintain athletes’ ankle proprioception during the late stages of a half-marathon (Chang et al., 2022). To date, however, little research has investigated the effect of compression garments on knee proprioception in half marathon runners. Such research may provide useful information for the use of compression garments in knee injury prevention during endurance running.

In addition to the possible impact of CG on proprioception, it is also unclear if they have any effect on cumulative fatigue in half-marathon running. CG have been reported to help the muscles maintain a similar power output while recruiting fewer motor units, which has a positive effect on the fatigue induced by prolonged muscle contraction (Fu and Xiong, 2009). Previous studies have utilized heart rate, RPE and blood lactic acid as physiological indicators of athletes’ level of fatigue (Hausswirth et al., 2000; Mizuno et al., 2017b). However, Chang et al. (2022) found that wearing graduated compression socks had no effect on these physiological measures. This might be because compression socks only cover the foot and ankle complex without large muscle groups being involved. In comparison, compression running pants (CRP) cover and compress the majority of the lower limb muscles. Currently, there is also insufficient evidence regarding the influence of CRP on physiological indicators of level of fatigue during long distance running.

Accordingly, the objectives of the present study were to determine whether wearing CRP and different running stages would affect knee proprioception and fatigue-related physiological responses during a half-marathon distance run on a treadmill, and to evaluate the association between knee proprioceptive acuity and fatigue-related physiological responses in half-marathon runners. We hypothesized that (1) During a treadmill prolonged running, compression running pants would maintain knee proprioceptive acuity and reduce fatigue-related physiological response seen in heart rate, RPE and blood lactic acid; and (2) fatigue-related physiological response would correlate with knee proprioceptive performance.

## Methods

### Sample size calculation

Sample size was calculated by the Gpower software for 80% power with an expected effect size of 0.30 and significance level of 0.05. Based on these values, 18 participants were recruited for the study.

### Research design

This study was approved by the Research Ethics Committee (102772020RT069). Each volunteer provided informed consent before participating.

**FIGURE 1:**
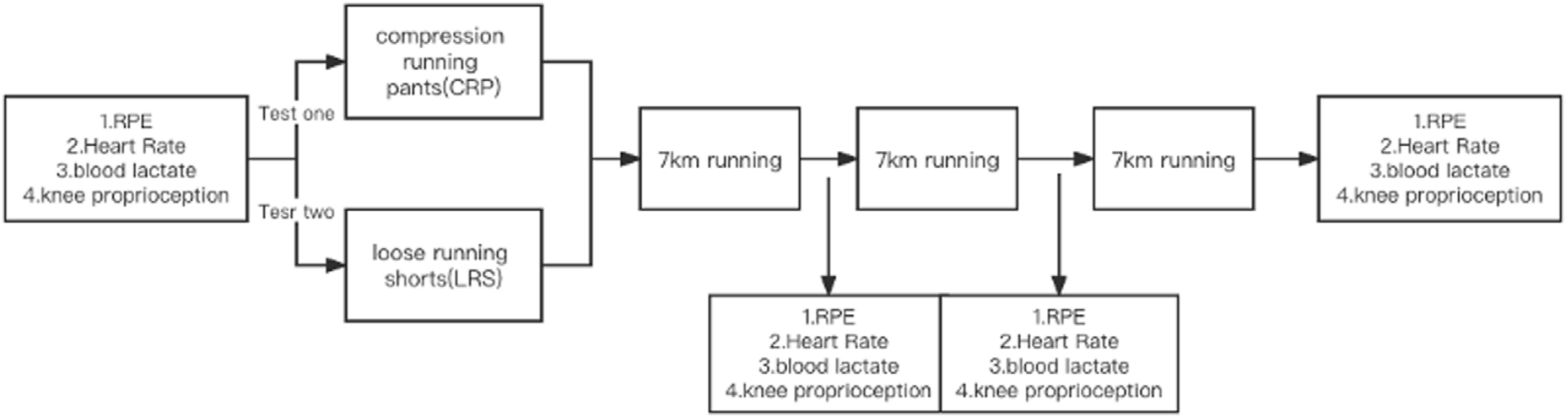
The experimental process. The study included two trials, in which each participant ran 21 km. The total distance of each run was 21 km, broken into three 7 km stages. Heart rate, RPE, and blood lactic acid were monitored, and knee flexion proprioception was tested for each participant over the stages of the run.

The study included two trials, in which each participant ran 21 km twice with a 1-week separation. Both running trials were performed on a laboratory treadmill at 14°C throughout to eliminate the influence of temperature. The total distance of each run was 21 km, broken into three 7 km stages (Gattoni et al., 2021). Heart rate, RPE, and blood lactic acid were monitored for each participant over the stages of the run, and knee flexion proprioception was tested before running began and subsequently after each 7 km immediately (Figure 1). The sequence of the two garment conditions was determined randomly. The random testing sequence was generated using Visual Studio Code software (https://code.visualstudio.com/, USA, Version 1.67) as follows: Import random/x = [1,2]/#1 represent CRP, 2 represent LRS/random. shuffle (x)/print(x).

### Participants

Eighteen participants aged 18 to 45 (mean = 35.3 ± 8.5 years) volunteered. Inclusion criteria for the participants were: (1) never used CRP before; (2) no history of lower extremity trauma or injury in the past 6 months; (3) regular half marathon training habits in the past 6 months. Participants were excluded if they had previous surgeries or any musculoskeletal injuries in the lower extremity. The participants’ demographics are reported in Table 1.

**TABLE 1:** Participant demographics: Mean (standard deviation).

	Male (N = 9)	Female (N = 9)	Total (N = 18)
Age (years)	32.0 (8.6)	38.7 (7.3)	35.3 (8.5)
Height (cm)	175.7 (7.2)	164 (8.7)	169.8 (9.8)
Weight (kg)	67.6 (8.1)	53.4 (4.9)	60.5 (9.8)
BMI (kg·m^−2^)	21.8 (1.9)	20.5 (1.5)	21.2 (1.8)
Training experience (years)	4.2 (2.2)	5.3 (4.8)	4.8 (3.6)
Weekly volume (km)	47.2 (23.7)	38.3 (21.1)	42.8 (22.2)
21 km best time	98.33 (5.8)	108.3 (5.4)	102.8 (7.8)

### Garments

Loose running shorts (LRS) were those that applied no pressure on the runner’s lower limbs. Compression running pants (CRP, NIKE Pro, made in Shanghai, China) were used as the intervention. The materials are 88% polyester fiber and 12% spandex. According to the manufacturer’s information, compression running pants are progressive, with calf pressure values ranging from 15 to 22 mmhg and thigh pressure values ranging from 12 to 20 mmhg. Each participant received the appropriate CRP based on the manufacturer’s sizing chart, to provide firm but comfortable compression.

### Measurements

At present, the markers for determination of exercise fatigue are mainly divided into the following: biochemical markers, physiological markers and psychological markers (Rietjens et al., 2005). Blood lactic acid is one of the biochemical markers commonly used to monitor exercise fatigue (Theofilidis et al., 2018). Secondly, among physiological markers, several studies have used heart rate as a fatigue evaluation marker (Jones and Doust, 1996; Goh et al., 2011; Mizuno et al., 2017a). In addition, RPE, as one of the psychological markers, is a good marker for monitoring the level of fatigue during prolonged exercise (Hausswirth et al., 2000). So, we selected blood lactate, heart rate and RPE as the biochemical markers, physiological markers and psychological markers from considerations of economy and convenience. Participants rated their perceived exertion (RPE) using the Borg scale (6–20) and during trials their heart rate was monitored continuously by the Polar H10 (Finland). The 6–20 Borg (1998) scale was used because there is a high correlation between the scale and heart rate, and the actual heart rate could be predicted. Combined with the actual heart rate measured with a Polar heart rate monitor in this study, the results could be more accurate. Blood lactic acid as an additional indicator of fatigue was assessed using the Lactate Scout4 (Germany).

Knee flexion proprioceptive acuity was assessed by the active movement extent discrimination apparatus (AMEDA) (Waddington and Adams, 1999; Waddington et al., 2000). A previous study has shown that the dominant side has poorer proprioceptive ability than another side (Han et al., 2013). Therefore, a main consideration in this study was injury prevention, we chose to test the joint on the poorer side for proprioception as a conservative representation of the participant’s proprioceptive ability. Proprioception of the dominant knee was assessed and the dominant side was determined by the Chinese version of the Waterloo Footedness Questionnaire (Yang et al., 2018).

### The active movement extent discrimination apparatus

**FIGURE 2:**
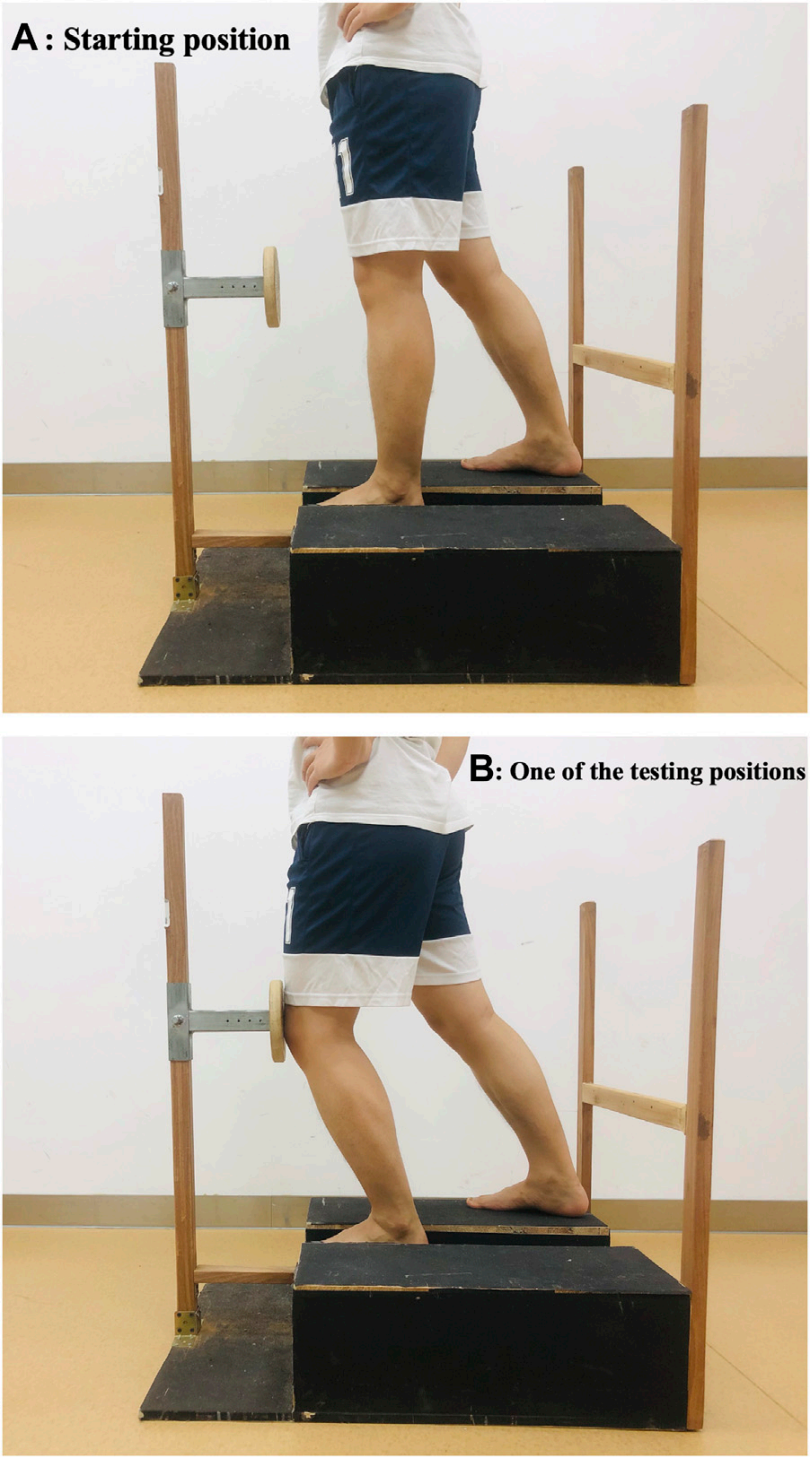
Knee proprioception assessed by the knee AMEDA. **(A)** Starting position: participants were instructed to look forward and perform a lunge squat from a standardized start position. **(B)** One of the testing positions: participants were instructed to make an absolute determination of knee lunge extent when their patella touched the wooden disc, using numbers 1,2,3 or 4.

The Knee AMEDA consists of three parts: a standing platform, a horizontal bar (A) parallel to and 20 cm away from the platform, and a moveable wooden disc with a radius of 10 cm (Figure 2). A rectangular groove with a depth of 5 cm is in the middle of the standing platform, and the participants’ test leg was positioned in this groove facing the moveable wooden disk. The height of the wooden disk was adjusted to the same level as the center of the participant’s patella. The distance between the wooden disc and the patella can be adjusted by the experimenter moving the wooden disc to produce 4 different distances from the midpoint of the patella, marked at position 1, 2, 3 and 4, with 1 = 11 cm, 2 = 12 cm, 3 = 13 cm and 4 = 14 cm. During the test, participants were instructed to look forward and perform a lunge squat from a standardized start position (Figure 2A) till their patella touched the wooden disc (Figure 2B), then they returned to the starting position.

The participant was initially familiarized with the four possible knee flexion positions by experiencing each of them in order, for of three rounds. During testing, the four possible knee flexion positions were randomly presented, ten times for each position. Participants were instructed to make an absolute determination as to which position (1,2,3 or 4) they had experienced each time (Figure 2).

### Data analysis

Acquired data were analyzed using IBM SPSS 24 (https://www.ibm.com/cn-zh/analytics/spss-statistics-software, United States), with the significance level set at 0.05. Knee flexion proprioceptive acuity was represented by the mean AUC score over adjacent positions, i.e., the Area Under the Receiver Operating Characteristic Curve (ROC), to indicate a participant’s proprioceptive discrimination sensitivity across the 4 different knee flexion positions (Hautus et al., 2021). The AUC value can range between 0.5 and 1.0, with the minimum value meaning chance responding and the maximum value meaning perfect discrimination.

The effects of CRP and different running stages on knee flexion proprioception, as well as any difference in heart rate, RPE and blood lactic acid between the two conditions over different running stages were examined using 2-way fully repeated measures ANOVA. The two repeated-measures factors were Garment Condition, with levels CRP and LRS, and Running Distance, with levels 0 km, 7 km, 14 km and 21 km. Polynomial trend analysis was applied to the factor Running Distance, with the 3 available degrees of freedom producing tests for linear (no turning points), quadratic (one turning point) and cubic (two turning points) components in the functions of the measure scores over running distance. The trend component weights and computational procedures are given in Winer (1991), Brown and Michels. This ANOVA analysis produces overall F-tests for Garment Condition, Linear Distance trend, Quadratic Distance trend, and Cubic Distance trend, as well as three interaction F-tests which address the questions of whether there are differences in the linear, quadratic and cubic components of the dependent variable functions between the occasions when runners were wearing either compression or loose shorts. Paired sample t-tests were carried out to investigate the differences between the two kinds of garments at the four different trial stages.

## Results

**FIGURE 3:**
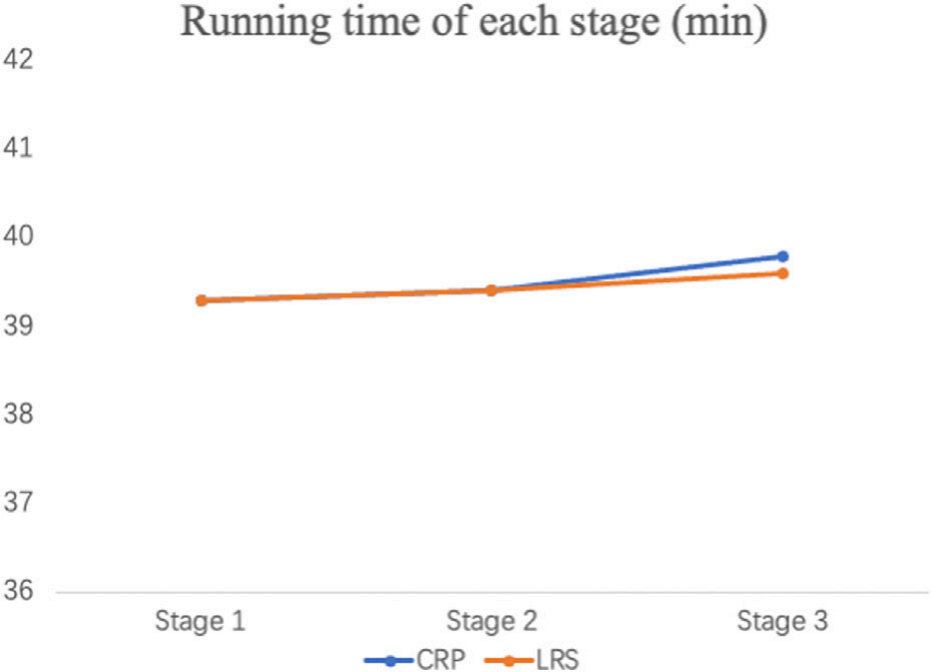
Running time for each stage in CRP and LRS.

**FIGURE 4:**
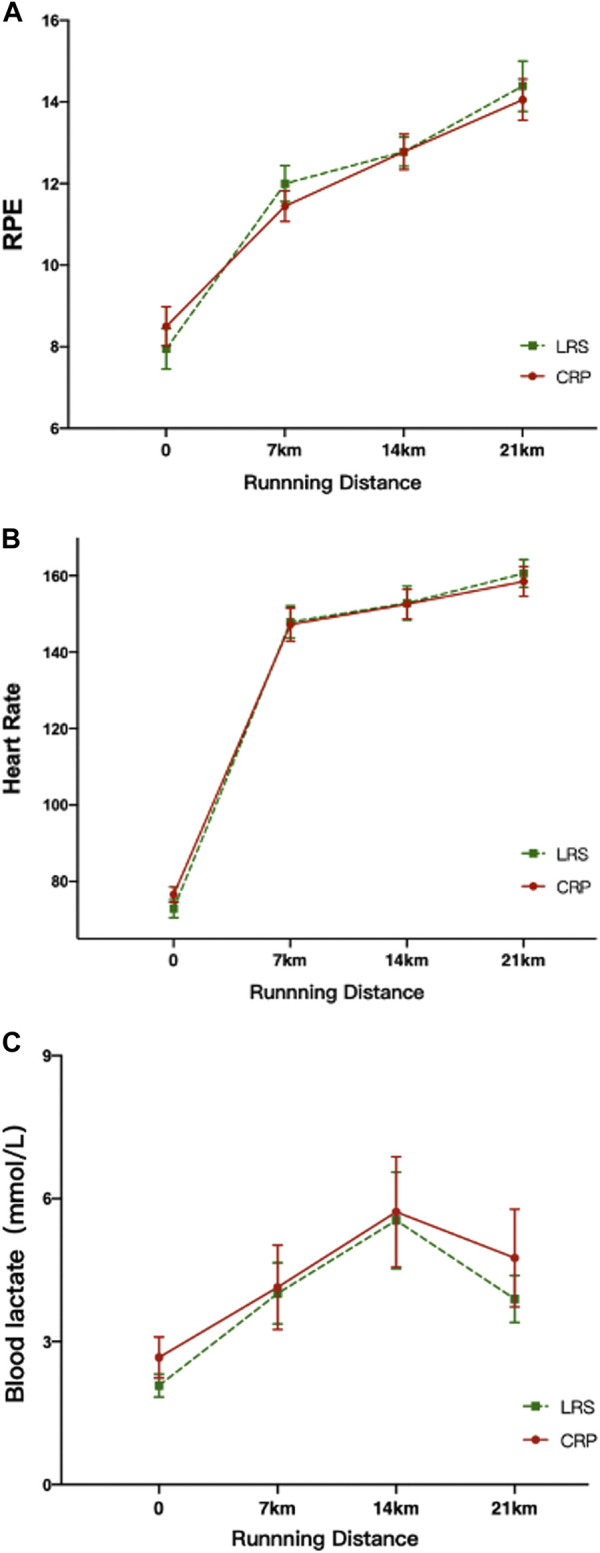
Heart rate, RPE and blood lactate at different running distances while wearing CRP and LRS. **(A)** RPE at different running distances; **(B)** Heart rate at different running distances; **(C)** blood lactate at different running distances. CRP, compression running pants; LRS, loose running shorts.

In the two garment running trials, participants ran at a self-paced speed, with the result being similar running times for CRP and LRS (mean ± SD: 118.9 ± 13.3, and 118.1 ± 13.5 min, respectively), with *p* = 0.156, 95% CI = −0.351, 2.018) (Figure 3). Repeated measures ANOVAs revealed no overall differences between CRP and LRS conditions in RPE (F_1,17_ = 0.211, *p* = 0.652), heart rate (F_1,17_ = 0.014, *p* = 0.907), or blood lactic acid (F_1,17_ = 0.619, *p* = 0.442), but wearing CRP was associated with better scores in AMEDA knee flexion proprioception (F_1,17_ = 9.936, *p* = 0.006). Trend analysis showed an overall significant linear increase with increased running distance in blood lactic acid (F_1,17_ = 7.788, *p* = 0.013), heart rate (F_1,17_ = 965.8, *p* < 0.001) and RPE (F_1,17_ = 91.3, *p* < 0.001)), but a significant linear decrease in AMEDA knee proprioception scores (F_1,17_ = 4.563, *p* = 0.048). With the fast, consistent initial rise in physiological measures over the first 7 km, there were significant quadratic components in the RPE, heart rate and blood lactic acid functions (F_1,17_ = 30.120, *p* < 0.001; F_1,17_ = 254.249, *p* < 0.001; F_1,17_ = 8.049, *p* = 0.011), but not for proprioception (*p* = 0.774). Significant cubic components were observed only with heart rate (F_1,17_ = 261.9, *p* < 0.001) and RPE (F_1,17_ = 20.1, *p* < 0.001), and are consistent with runners having adopted a “float” phase in the middle section between the rising initial and final sections. There were no interactions between Garment Condition and any of the trend components (all *p* > 0.05) with one exception, this being heart rate, where the higher heart rate upon application of CRP, and lower heart rate at 21 km, produced a significantly flatter slope for the linear trend component when wearing CRP (F_1,17_ = 5.731, *p* = 0.028). These findings show increasing fatigue during the two garment condition running trials, with a generally similar change pattern in fatigue measures and perceived exercise intensity observed in both, indicating that wearing CRP did not affect heart rate, RPE or blood lactic acid (Figures 4A–C). However, significantly better knee proprioception was observed when wearing CRP.

**FIGURE 5:**
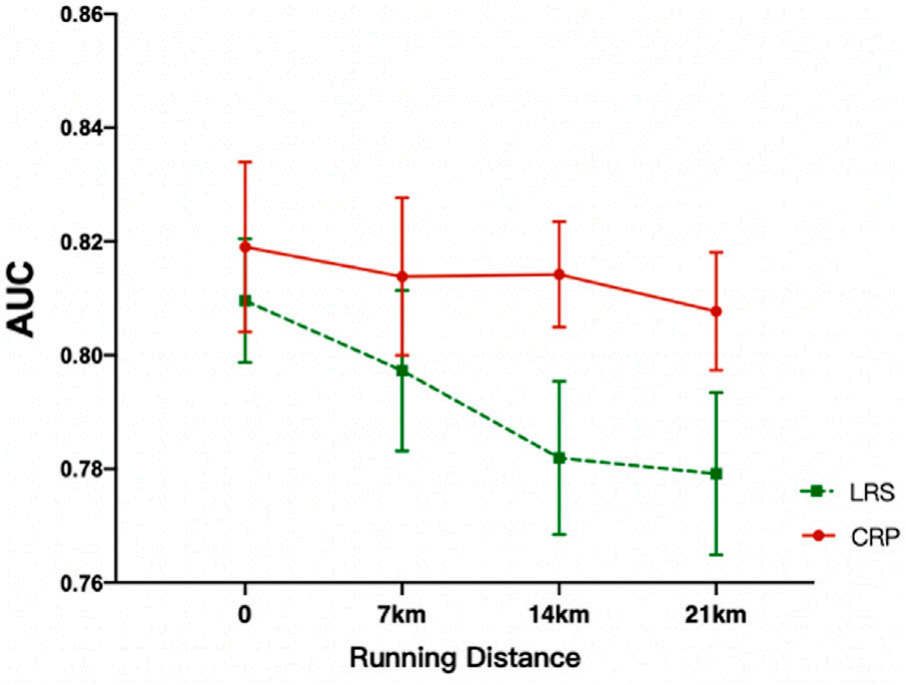
Knee flexion proprioceptive acuity at different running distances in the CRP and LRS conditions. CRP, compression running pants; LRS, loose running shorts. * = *p* < 0.05; AUC, area under the receiver operating characteristic curve.

Post-hoc analysis using paired sample t-tests, conducted to determine the location of simple effects, showed that there was no statistically significant difference in knee proprioceptive acuity between the two garment-wearing conditions at the beginning or after 7 km of running, but after 14 km, compared to the LRS condition, participants performed significantly better when wearing CRP after 14 km (*p* = 0.007, 95% CI:−0.054, −0.010) and this was also the case after 21 km (*p* = 0.016, 95% CI: −0.051, −0.006), (Figure 5 and Table 2).

**TABLE 2:** Difference of knee AUC between two conditions at different running stages (Mean ± SD).

Stage	CRP AUC	LRS AUC	Mean difference	*p*	95%C.I
1	0.819 ± 0.063	0.810 ± 0.046	−0.009 ± 0.066	0.550	−0.042, 0.023
2	0.814 ± 0.059	0.797 ± 0.060	−0.017 ± 0.042	0.110	−0.037, 0.004
3	0.814 ± 0.040	0.782 ± 0.057	−0.032 ± 0.045	0.007**	−0.054, −0.010
4	0.808 ± 0.043	0.780 ± 0.061	−0.029 ± 0.045	0.016*	−0.051, −0.006

* *p* < 0.05, ** *p*< 0.01. CRP, compression running pants; LRS, loose running shorts; SD, standard deviation; 1 = before running, 2 = after 7 km run, 3 = after 14 km run, 4 = after 21 km run.

## Discussion

In this study, RPE and heart rate increased during both 21 km running trials, indicating that for a group of trained half-marathon runners, fatigue gradually accumulated during the half marathon run in a pattern that was faster in the initial and later stages. Whether runners wore loose shorts or compression running pants had most effect on the measure of knee proprioception. While knee proprioception decreased significantly during the 21 km of treadmill running, wearing CRP had an overall beneficial effect on knee proprioceptive acuity, and in particular, maintained knee proprioception after runners had completed 14 km and 21 km. There was no difference in RPE, heart rate, or blood lactic acid level between the two garment conditions, suggesting that CRP had no effect on these fatigue-related physiological indicators.

CRP did not affect heart rate, RPE, or blood lactate level during running. Hsu et al. (2020) conducted a 40-min running experiment with participants wearing CRP and standard sports pants respectively and found that CRP did not change the participants’ RPE and blood lactic acid, which is consistent with results from the current study. These authors speculated that RPE may not be a sensitive indicator of any CRP effect in long-distance running. In contrast, wearing CRP showed a significant positive effect on RPE when running 15 min (Rugg and Sternlicht, 2013) and 400 m (Faulkner et al., 2013). Accordingly, it may be that CRP may have a positive effect on perceived exertion only in short distance running, but not in long distance running. Results here showed that heart rate increased during running the half marathon in both CRP and LRS conditions, although the rate of increase was significantly, albeit marginally, less in the CRP condition. Compression running pants have been thought to potentially aid muscle contraction and increase venous return (Ibegbuna et al., 2003) subsequently decreasing heart rate during constant speed exercise. However, there was no difference in heart rate observed here between the two conditions, indicating that venous return may not have improved, presumably because the participants would have had enough blood flowing from the legs to the heart during high-intensity exercise (Kuipers et al., 1989). In the present study, blood lactate was observed to increase continuously in the first and second stage but to decrease in the third 7 km stage. Previous studies have shown that for sustained exercise, blood lactate concentration usually increases at the beginning and then increases rapidly as the intensity of exercise increases (Goodwin et al., 2007; Hsu et al., 2020). It is possible that the time taken here to test knee proprioception, coupled with the knowledge that the third section was the final one, were sufficient to halt the normally expected rise in blood lactate concentration.

Wearing CRP maintained knee proprioceptive acuity at a higher level after 14 km and 21 km. It is hypothesized that this may be because the runner’s significantly increased stride length and significantly decreased stride frequency and peak knee flexion during swing after 8 km (Chan-Roper et al., 2012), indicative of fatigue. From this point, wearing CRP better maintained knee proprioception. In comparison, Chang et al. (2022) observed that graduated compression socks maintained ankle proprioception at the end of a 21 km run. This suggests that CRP may have an earlier positive effect on knee proprioception than ankle proprioception. This may be related to the first indication of fatigue in the biceps and rectus femoris during long-distance running (Chan-Roper et al., 2012), so that to a greater extent CRP may also reduce muscle vibration in larger muscle groups that contributes to fatigue and injury, thereby maintaining the knee proprioception. In addition, it is possible that CRP may cover more skin area and provide more skin sensory input, resulting in an earlier effect on knee proprioception.

The results of the present study indicate that the influence of wearing CRP on knee proprioception changes with running distance in half marathon runners, which suggests that training guidance can be given accordingly. Future studies might further refine the distance between 7 and 14 km to find the key point at which wearing CRP significantly improves proprioception under fatigue, to provide more accurate applications for prevention and intervention.

There are some limitations in this study: (1) participants conducted the whole process in an indoor environment and on a treadmill so as to control the influence of temperature, and the stability may not fully simulate a real marathon; (2) participants ran independently during the experiment, compared to having a crowd effect during the usual half marathon, and this may cause psychological differences and result in sports performance that cannot be completely replicated during a half marathon. Future research should focus on several people running on the road at the same time and try to control the environment as much as possible.

## Conclusion

Compared to wearing LRS, wearing CRP significantly elevated knee proprioceptive acuity, and maintained this improvement in after running 14 km and 21 km of a half-marathon. Furthermore, Compression running pants had no significant effect on fatigue-related physiology measures during running. These findings suggest that wearing CRP might prevent knee injuries related decreased knee proprioception in running the half marathon.

## Practical applications

• In the half marathon, knee proprioception decreases significantly with the increase in running distance but is elevated by wearing compression running pants. This finding has implications for athletes and practitioners concerned with injury prevention when running with fatigue.
• Compression running pants may reduce knee injury rates in half marathon running, as they are associated with better knee proprioceptive sensitivity at 14 km and 21 km.

## Data Availability

The original contributions presented in the study are included in the article/Supplementary Material; further inquiries can be directed to the corresponding author.
